# Prevention and Control of Zika as a Mosquito-Borne and Sexually Transmitted Disease: A Mathematical Modeling Analysis

**DOI:** 10.1038/srep28070

**Published:** 2016-06-17

**Authors:** Daozhou Gao, Yijun Lou, Daihai He, Travis C. Porco, Yang Kuang, Gerardo Chowell, Shigui Ruan

**Affiliations:** 1 Mathematics and Science College, Shanghai Normal University, Shanghai 200234, China; 2 Department of Applied Mathematics, The Hong Kong Polytechnic University, Hung Hom, Kowloon, Hong Kong, China; 3 Francis I Proctor Foundation, Department of Epidemiology and Biostatistics, and Department of Ophthalmology; University of California at San Francisco, San Francisco, CA 94143 USA; 4 School of Mathematical and Statistical Sciences, Arizona State University, Tempe, AZ 85287, USA; 5 School of Public Health, Georgia State University, Atlanta, GA 30302, USA; 6 Department of Mathematics, University of Miami, Coral Gables, FL 33146, USA

## Abstract

The ongoing Zika virus (ZIKV) epidemic in the Americas poses a major global public health emergency. While ZIKV is transmitted from human to human by bites of *Aedes* mosquitoes, recent evidence indicates that ZIKV can also be transmitted via sexual contact with cases of sexually transmitted ZIKV reported in Argentina, Canada, Chile, France, Italy, New Zealand, Peru, Portugal, and the USA. Yet, the role of sexual transmission on the spread and control of ZIKV infection is not well-understood. We introduce a mathematical model to investigate the impact of mosquito-borne and sexual transmission on the spread and control of ZIKV and calibrate the model to ZIKV epidemic data from Brazil, Colombia, and El Salvador. Parameter estimates yielded a basic reproduction number 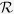
_0_ = 2.055 (95% CI: 0.523–6.300), in which the percentage contribution of sexual transmission is 3.044% (95% CI: 0.123–45.73). Our sensitivity analyses indicate that 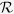
_0_ is most sensitive to the biting rate and mortality rate of mosquitoes while sexual transmission increases the risk of infection and epidemic size and prolongs the outbreak. Prevention and control efforts against ZIKV should target both the mosquito-borne and sexual transmission routes.

Zika virus (ZIKV), a *Flavivirus* closely related to dengue, is primarily transmitted to humans by the bites of infected female mosquitoes from the *Aedes* genus. These mosquitoes, widespread in tropical and subtropical regions, also transmit dengue fever, chikungunya, yellow fever, and Japanese encephalitis. For ZIKV, about one in five infected people develops symptoms including mild fever, rash, conjunctivitis and joint pain, with no documented fatalities seen in a recent large outbreak^1^. There is evidence that ZIKV increases the chances of microcephaly in newborn babies of infected mothers^2^,^3^ and some evidence suggests that it causes Guillain-Barré syndrome (GBS) as well^4^. Unfortunately, no vaccine, specific treatment, or fast diagnostic test is available to treat, prevent, or diagnose ZIKV infection at this time.

The virus was initially isolated from a rhesus monkey in the Zika forest of Uganda in 1947 and later isolated from humans in Nigeria in 1954^5^,^6^,^7^. Subsequently, only sporadic confirmed human cases were reported from Africa and Southeast Asia. In April 2007, the first documented ZIKV outbreak outside traditionally affected areas occurred on Yap Island, Federated States of Micronesia, in the North Pacific^1^. In October 2013, a severe ZIKV outbreak was reported in French Polynesia, South Pacific, with an estimated 28,000 cases^8^. The ongoing outbreak, which began in April 2015 in Brazil, has rapidly spread to many other countries in South and Central America and the Caribbean with more than 140,000 suspected and confirmed cases by the end of February 2016^9^. Nearly 6,000 suspected cases of microcephaly (including 139 deaths) among newborns might be linked to ZIKV infections in Brazil between October 2015 and February 2016. From December 2015 to February 2016, more than 200 GBS cases with history of suspected ZIKV infection were recorded in Colombia and 118 GBS (including 5 deaths) cases were reported in El Salvador^9^. The WHO declared the epidemic a Public Health Emergency of International Concern (PHEIC) on February 1, 2016^10^, and the U.S. CDC’s Emergency Operations Center has moved to the highest level of activation on February 3, 2016^11^. Based on the reported dengue data from 2015, WHO estimated that up to four million people in the Americas could be infected by ZIKV in 2016. Without effective intervention, the situation has considerable potential to worsen, due in part to the upcoming 2016 Summer Olympics in Rio de Janeiro as well as anticipated mosquito abundance increases caused by an ongoing El Niño.

ZIKV has been detected in serum, saliva, urine, and semen^12^,^13^,^14^. It has also been detected in urine and semen even after it disappears from blood^15^, and in one convalescent case it was detected in semen 27 and 62 days after onset of febrile illness^14^. Indeed, recent studies show that ZIKV can be transmitted via sexual contact. In 2011, it was reported that an infected male had infected a female by having vaginal sexual intercourse, even before his onset of symptoms^16^. After the confirmation of the first case of sexually transmitted ZIKV of the current outbreak in Dallas County by the CDC on February 2, 2016^17^, six more confirmed and probable cases of sexual transmission of ZIKV in the U.S. were reported by CDC on February 26, 2016^18^, and Europe’s first case of sexually transmitted ZIKV was diagnosed in France in Febraury 2016^19^. A case of ZIKV infection imported in Florence, Italy ex-Thailand, leading to a secondary autochthonous case, probably through sexual transmission in May 2014 was retrospectively diagnosed in 2016^20^. Since 2015, Zika infections likely acquired through sex have been reported in Argentina, Canada, Chile, France, Italy, New Zealand, Peru, Portugal, and the USA^21^.

The study of the ZIKV outbreak on Yap Island^1^ indicates that cases occurred among all age groups, but the incidence of ZIKV disease was highest among persons 55 to 59 years of age with the mean age of 36 years and 61% female. Since ZIKV infections are mostly asymptomatic or have mild symptoms lasting two to seven days, the disease has little impact on sexual activity^18^. If ZIKV is sexually transmissible, then it is necessary to abstain from sexual activity or consistently use condoms during convalescence. CDC has recently issued interim guidance on safe sex during a Zika outbreak^22^. This is particularly important to pregnant women in areas where the ZIKV is circulating.

## Results

### Modeling

**Figure 1:**
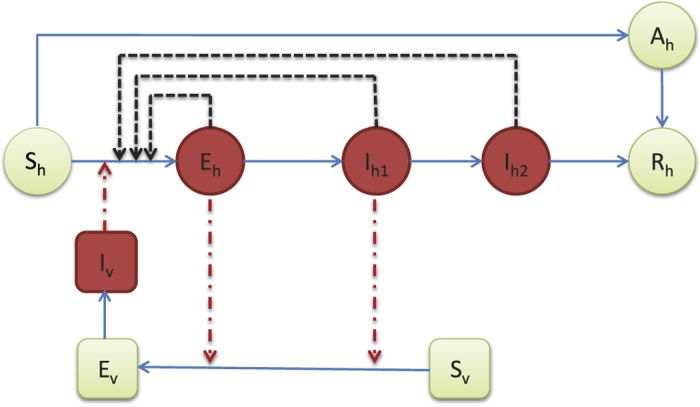
Flow diagram for the spread of ZIKV involving vectorial and sexual transmission. Green nodes are non-infectious and red nodes are infectious. Blue solid arrows show the progression of infection. Black dashed arrows show direction of human-to-human transmission and red dash-dotted lines show direction of transmission between humans and mosquitoes. An individual may progress from susceptible (*S*_*h*_) to asymptomatically infected (*A*_*h*_) to recovered (*R*_*h*_), or exposed (*E*_*h*_) to symptomatically infected (*I*_*h*1_) to convalescent (*I*_*h*2_) to recovered (*R*_*h*_), while a mosquito may progress from susceptible (*S*_*v*_) to exposed (*E*_*v*_) to infectious (*I*_*v*_).

Mathematical modeling has become a crucial tool in designing prevention and control measures for infectious diseases^23^,^24^. A recent study^25^ used a mosquito-borne model to examine the 2013–14 outbreak of Zika on the six major archipelagos of French Polynesia. To investigate the role of sexual transmission in the spread and control of Zika virus disease, we developed a deterministic model of Zika disease transmission that takes into account both mosquito-borne and sexual transmission modes (Fig. 1). Symptomatically infected humans are contagious to both mosquitoes and humans during the incubation period that is typically between 2 and 7 days. This is because the viremia and virusemenia occur before the end of the incubation period, although the viral load of exposed (presymptomatic) people may be lower^16^. After this period, infected humans develop symptoms. Symptomatic humans are more contagious to mosquitoes than exposed individuals and are also able to transmit the virus to partners through sex^18^. The virus appears to persist longer in semen and urine than in serum^14^,^15^. Following the period of viremia, symptomatic humans enter the convalescent stage and can no longer infect mosquitoes. However, such individuals remain infectious to humans, though with reduced infectivity. The infected humans’ convalescent period ends with lifelong immunity. Sexual transmission of ZKIV from asymptomatically infected humans has not been documented, so they are assumed to be noninfectious to humans. The timescale of human demography is far longer than that of the epidemiological dynamics, so we ignore human births and deaths when modeling an outbreak.

We make the following additional assumptions: (i) Mosquitoes cannot be infected by biting asymptomatically ZIKV infected people; (ii) The sexual ratio of humans is 1:1 and male and female are subject to almost the same epidemiological factors; (iii) The end of the viremic period coincides with the disappearance of symptoms in symptomatically infected individuals (see Fig. 1).

### Parameter estimates

All parameter descriptions and ranges are summarized in Table 1. To parameterize our model, we used reasonable epidemiological parameters based on our current understanding of Zika epidemiology and transmission dynamics. Zika virus and dengue virus are arboviruses of the same genus *Flavivirus* spread by mosquitoes of the same genus *Aedes* and have similar symptoms, high proportion of asymptomatic infections, duration of incubation and infectiousness^7^. Hence they have the same number of bites on humans per mosquito per unit time, *a*, and we anticipated that their transmission probabilities per bite from mosquitoes to humans, *b*, and from humans to mosquitoes, *c*, respectively, are comparable.

Many parameters of ZIKV infection are not available, but we can make some reasonable assumptions to estimate specific measurements of the infectivity. For instance, consideration of other human sexually transmitted infections may provide an initial basis for analysis. We note that gonorrhea appears to display a high probablity of transmission per coital act (results ranging from 0.19–0.65, with male to female transmission higher than female to male^26^). Others show a considerably lower transmission probability per coital act, such as HSV-2 (0.0005)^27^.

The average sexual frequency over sexually active ages is twice a week and the frequency of sexual intercourse over all age groups is assumed to be once a week^28^. The sexual transmission rate of symptomatically infected people (transmission probability × contact rate), *β*, is assumed to range from 0.001 to 0.10, which means the transmission probability per sex act is between 0.007 (mild infectivity) and 0.70 (severe infectivity).

### Fitting Zika data in Brazil, Colombia and El Salvador

**Figure 2:**
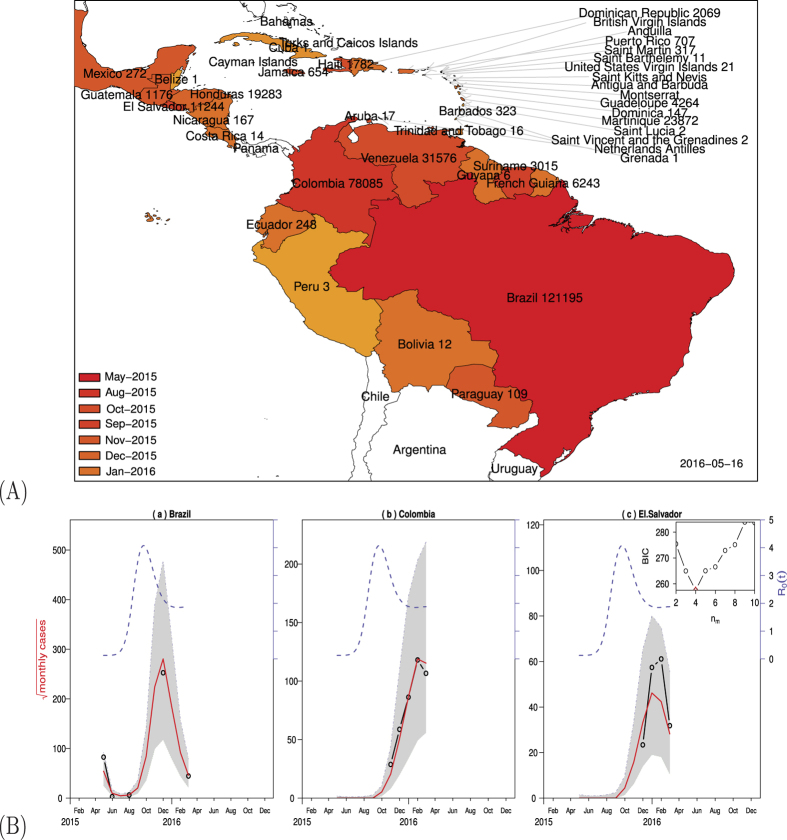
(**A**) ZIKV outbreaks in South and Central Americas. The map indicates the month of first reported cases and the cumulative cases by May 16, 2016, in each country. The map was made with the free software “R: A Language and Environment for Statistical Computing, R Core Team, R Foundation for Statistical Computing, Vienna, Austria (2016) https://www.R-project.org.” accessed on February 1, 2016. (**B**) Fitting model to data in Brazil, Colombia, and El Salvador up to February 27, 2016. Each panel shows the simulation (red solid curve) versus the observed (black circle), with the best fitting parameters. The red solid curves show median values of 1000 simulations and shaded region show the 95% range. The blue dash curves show the estimated mosquito-human population ratio *m*(*t*). The inset panel shows Bayesian Information Criterion (BIC) as a function of the number of nodes (*n*_*m*_) in *m*(*t*) with values *m*_*i*_ at these nodes. Assumed or estimated parameters and initial conditions are given in Table 2.

To use our model to fit the reported ZIKV cases in Brazil, Colombia, and El Salvador (see Fig. 2(A)), we assumed that the three countries share common parameter values (see Table 1), except for country population size and initial conditions (see Table 2). Since large scale mosquito-control campaign has been taken in these Zika affected countries, we assumed that the ratio of mosquitoes to humans *m* is time-dependent and used a cubic spline function of time with *n*_*m*_ parameters to describe *m*(*t*).

Figure 2(B) demonstrates that our model provides good fits to the reported Zika data from Brazil, Colombia, and El Salvador up to February 27, 2016. Since *m*(*t*) is time-depedent, so is 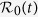 which is represented by the right vertical axis. In Brazil, the outbreak started in the spring of 2015, has passed its peak, and seems under control for the time being. In Colombia and El Salvador, the disease started in the summer of 2015 and is reaching its peak now. More Zika, GBS and microcephaly cases are expected from other countries in South and Central Americas and the Caribbean. The starting time and geographic spread of Zika (Fig. 2(A)) indicates that it is following the path of dengue and chikungunya and has the potential to be introduced to many other countries where the *Aedes* species mosquitoes are competent, including some southern states in the U.S.

### Estimation of the basic reproduction number

**Figure 3:**
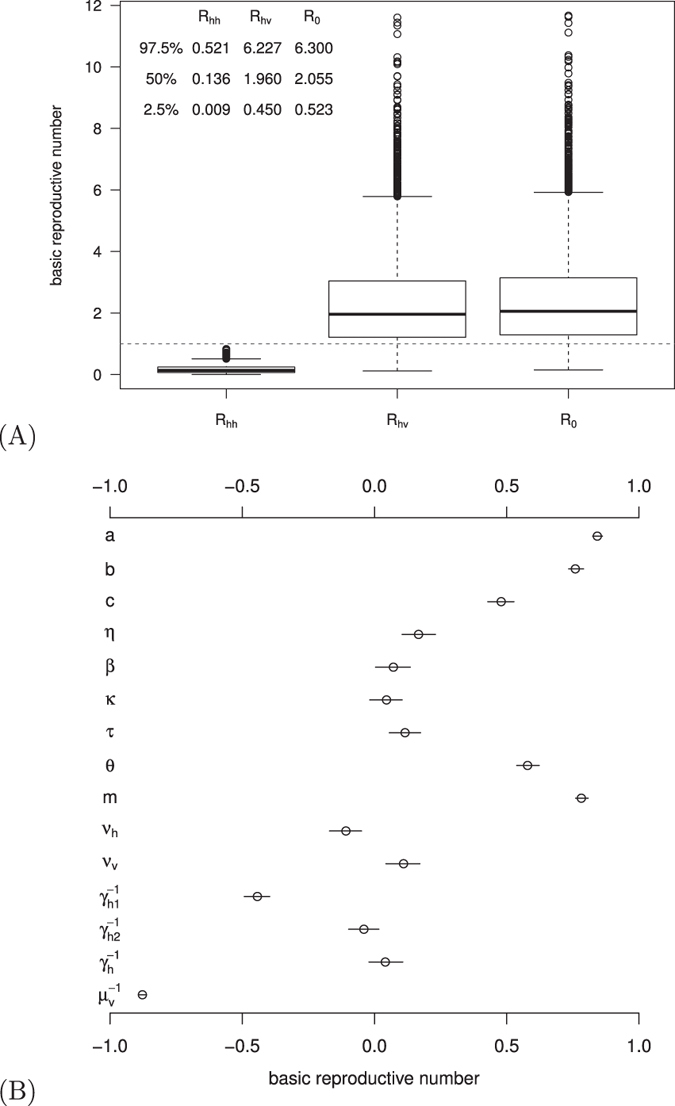
(**A**) The box plot of the basic reproduction numbers for one or both transmission routes. The horizontal dashed line is 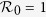. (**B**) The partial rank correlation coefficient (PRCC) of the basic reproduction number with respect to model parameters. The circle is the estimated correlation and the bar shows the 95% confidence interval. Parameter ranges are given in Table 1.

Based on parameter ranges in Table 1, we used the Latin hypercube sampling method^29^ to generate 5,000 samples by assuming a uniform distribution for each parameter, and calculated the corresponding uncertainty on the basic reproduction numbers of either mosquito-borne transmission or sexual transmission or both. The median and confidence interval of the distribution of the basic reproduction numbers (see Fig. 3(A)) are 2.055 (95% CI: 0.523–6.300) for 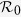, 1.960 (95% CI: 0.450–6.227) for 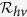, and 0.136 (95% CI: 0.009–0.521) for 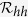, respectively; the median and confidence interval of the distribution of the percentage of contribution by sexual transmission in 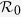 is 3.044 (95% CI: 0.123–45.73). This suggests that sexual transmission alone is unlikely to initiate or sustain an outbreak. However, if the human-to-human transmission probability is very high, then its promoting effect on the transmission of ZIKV cannot be neglected.

To identify the key factors that determine the magnitude of the basic reproduction number, we performed global sensitivity analysis (see Fig. 3(B)) with 1,000 random sample uniformly distributed in the range of parameter values from Table 1 and 1,000 bootstrap replicates^30^. The basic reproduction number is most sensitive to mosquito biting rate and mortality rate.

**Figure 4:**
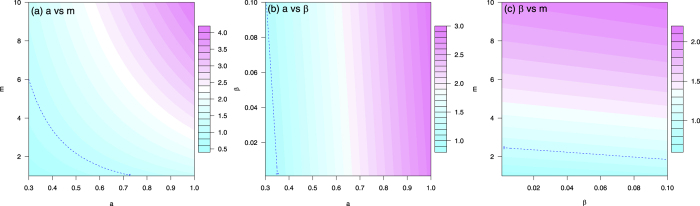
The contour plot of the basic reproduction number in terms of two of the three controllable parameters: *β* (transmission rate of symptomatically infected humans to susceptible humans), *m* (ratio of mosquitoes to humans), and *a* (mosquito biting rate). The blue dashed curve is the contour of 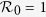. Parameter values are given in Table 1.

To determine the dependence of the basic reproduction number on the controllable model parameters, choose *b* = 0.4, *c* = 0.5, *η* = 0.1, *κ* = 0.6, *τ* = 0.3, *θ* = 0.18, *ν*_*h*_ = 1/5, *ν*_*v*_ = 1/10, *γ*_*h*1_ = 1/5, *γ*_*h*2_ = 1/20, *γ*_*h*_ = 1/7, *μ*_*v*_ = 1/14, and the three controllable parameters *β* = 0.05 or in (0.001, 0.10), *m* = 5 or in (1, 10), *a* = 0.5 or in (0.3, 1). Fixing one of *β*, *m*, and *a* at the specific value and varying the other two parameters, the contour plots of 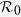 in terms of *a* and *m* (left panel), *a* and *β* (middle panel), *β* and *m* (right panel) are illustrated in Fig. 4, respectively. The basic reproduction number ranges from 0.5 to 4, the disease cannot spread if 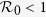 and can cause an outbreak otherwise.

Figure 4 indicates that mosquito-control, personal biting protection, and sexual contact protection are all important measures to control Zika virus infection. As season changes, *m* will change along time which may cause fluctuating outbreaks of Zika in the future. Note that even the vector-control and bitting protection measures are successful, if the human-to-human sexual transmission probablity is very high, then the disease still could persist in the population and the outbreak could be prolonged.

### Attack Rate

Attack rate or attack ratio *z* is the fraction of the population that becomes infected, which is also an important concept in measuring the transmission of infectious diseases.

**Figure 5:**
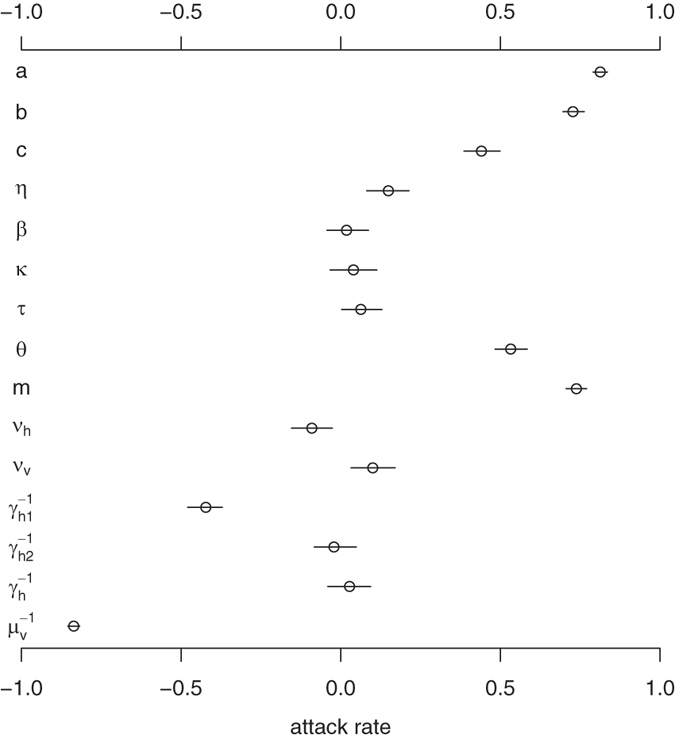
The partial rank correlation coefficient (PRCC) of the attack rate with respect to model parameters. The circle is the estimated correlation and the bar shows the 95% confidence interval. Parameter ranges are given in Table 1.

To identify the key factors that affect the attack rate, we performed global sensitivity analysis (see Fig. 5) with 1,000 random sample uniformly distributed in the range of parameter values from Table 1 and 1,000 bootstrap replicates. The attack rate is also most sensitive to mosquito biting rate and mortality rate.

**Figure 6:**
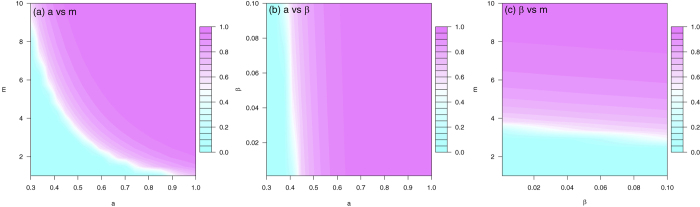
The contour plot of the attack rate in terms of two of the three controllable parameters: *β* (transmission rate of symptomatically infected humans to susceptible humans), *m* (ratio of mosquitoes to humans), and *a* (mosquito biting rate). All parameter values are given in Table 1.

To determine the dependence of the attack rate on the controllable model parameters, choose *b* = 0.4, *c* = 0.5, *η* = 0.1, *κ* = 0.6, *τ* = 0.3, *θ* = 0.18, *ν*_*h*_ = 1/5, *ν*_*v*_ = 1/10, *γ*_*h*1_ = 1/5, *γ*_*h*2_ = 1/20, *γ*_*h*_ = 1/7, *μ*_*v*_ = 1/14, and the three controllable parameters *β* = 0.05 or in (0.001, 0.10), *m* = 5 or in (1, 10), *a* = 0.5 or in (0.3, 1). Fixing one of *β*, *m*, and *a* at the specific value and varying the other two parameters, the contour plots of the attack rate *z* in terms of *a* and *m* (left panel), *a* and *β* (middle panel), and *β* and *m* (right panel), are illustrated in Fig. 6, respectively.

Based on the attack rate 73% (95% CI: 68–77%) observed in the Yap Island ZIKV outbreak of 2007^1^, we estimated the basic reproduction number according to the final size formula 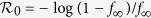 in the absence of behavior changes, control interventions, and so on, where *f*_∞_ is the final infected fraction^31^,^32^. These yield 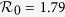 with uncertainty interval 1.68–1.91. We further note that with 11% of the population seeking medical care for suspected ZIKV infection during the French Polynesia ZIKV outbreak of 2013^8^, consistent with a total attack fraction over 0.55 and 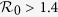 (serological surveys of the attack fraction in French Polynesia are ongoing). These estimates are roughly consonant with the value of 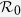 (1.6–2.5) for dengue in Brazil^33^. Of course, the estimate of 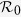 was obtained by assuming random mixing and a constant vector population over the course of the epidemic. Extrapolation of the attack rate seen in the Yap Island outbreak would be potentially misleading, due to differences in vectorial capacity, heterogeneity in space, as well as vector control and infection minimization strategies (e.g., bednets) that would occur during larger scale epidemics.

### Numerical Scenarios

In Table 3, selected scenarios from Fig. 4 are presenteded, demonstrating the range of transmission contributed by sexual activity. If *m* = 5 and *a* = 0.5, sexual transmission only accounts for 1.351% of total infections at *β* = 0.01, increases to 6.506% if *β* = 0.05 and 12.45% if *β* = 0.10. When *β* = 0.05, *m* = 5, *a* = 0.5, the attack rate is up to 82.65%; it is impossible to contain infection even reducing mosquito population by 55%, though the reproduction number for human-mosquito transmission is less than one in this scenario. In this case, both public and individual disease intervention measures are imperative (e.g., 55% reduction in mosquito population and 50% reduction in unprotected sex behaviors). In all scenarios, *b* = 0.4, *c* = 0.5, *η* = 0.1, *κ* = 0.6, *τ* = 0.3, *θ* = 0.18, *ν*_*h*_ = 1/5, *ν*_*v*_ = 1/10, *γ*_*h*1_ = 1/5, *γ*_*h*2_ = 1/20, *γ*_*h*_ = 1/7, *μ*_*v*_ = 1/14. The three columns refer to controllable parameters, basic reproduction numbers, and attack rates, respectively.

## Discussion

Lacking data on early dynamics to dissect the contributions from different transmission routes of ZIKV, we designed a SEIR-type model based on classic epidemic theory^23^ and studied the impact of sexual transmission on the prevention and control of the mosquito-borne ZIKV. As a result, we considered here the exponential growth dynamics of ZIKV infection. In contrast, sub-exponential growth dynamics for sexually-transmission diseases has been reported previously^34^. By using reasonable ranges of parameter values, we obtained some estimates of the basic reproduction number and provided a qualitative analysis of the contribution of mosquito-borne transmission and sexual transmission on the spread of ZIKV.

Sexual transmission increases the risk of infection and epidemic size, but itself may not initiate or sustain an outbreak. Statistically, the transmission contributed by sexual activity is a small percentage of the total transmission in attack rate (4.437 (95% CI: 0.297–23.02)). However, the potential of non-vector-borne transmission could complicate efforts on containing the spread of ZIKV. Under certain circumstances, culling a large fraction of mosquito population such that the basic reproduction number for human-mosquito transmission below one may be not sufficient in the absence of sexual risk reduction.

At the beginning of an outbreak, it is best to contain ZIKV infection by vector control (larviciding and adulticiding) and insect bite precautions (bed net and insect repellents). However, insecticide resistance in *Aedes aegypti* and *Aedes albopictus* and poverty in the most vulnerable regions can compromise the success of bite reduction. Subsequently, measures on reduction of sexual transmission shall be implemented. Especially, pregnant women or those at childbearing age who have pregnancy planning need to avoid unprotected sexual contact.

To better understand the transmission mechanisms and estimate the level of sexual transmissibility, solid clinical and epidemiological data are required. However, the cross-reaction of other *Flaviviruses* and the mild illness of Zika make the infections highly unreported or misdiagnosed. The magnitude of human-to-human transmission may be underestimated if a longer convalescent phase with higher infectivity is confirmed, or overestimated if infected women cannot transmit Zika virus to their sex partners. We assumed a homogeneous mixing human population, although heterogeneities such as gender, culture, religion, and socioeconomics deserve further investigation. Moreover, it is much more difficult to formulate, parametrize, validate and analyze such models.

If sexual transmission were not small compared to vector-borne transmission, there would have been some excess seen in particular age groups (sexually active age^1^), partnership groups (individuals with regular sexual intercourse), behavioral groups (individuals frequently having unprotected sex), and professional groups (sex workers). A standard questionnaire plus a recent history of sexual contacts for residents in Zika-hit regions will provide these data to examine the mode of sexual transmission.

## Methods

### Mathematical Model

Human population is divided into six classes: susceptible (*S*_*h*_(*t*)), exposed (*E*_*h*_(*t*)), symptomatically infected (*I*_*h*1_(*t*)), convalescent (*I*_*h*2_(*t*)), asymptomatically infected (*A*_*h*_(*t*)), and recovered (*R*_*h*_(*t*)) at time *t* > 0, and the mosquito population is divided into three classes: susceptible (*S*_*v*_(*t*)), exposed (*E*_*v*_(*t*)), and infectious (*I*_*v*_(*t*)), respectively. *N*_*h*_ = *S*_*h*_ + *E*_*h*_ + *I*_*h*1_ + *I*_*h*2_ + *A*_*h*_ + *R*_*h*_ denotes the total number of humans and *N*_*v*_ = *S*_*v*_ + *E*_*v*_ + *I*_*v*_ denotes the total mosquito population, both are assumed to be constant. We use the SEI type of structure for mosquitoes and SEIR type of structure for humans^23^,^24^. Based on the assumptions, the ZIKV transmission dynamics between humans and mosquitoes are governed by the following model equations:


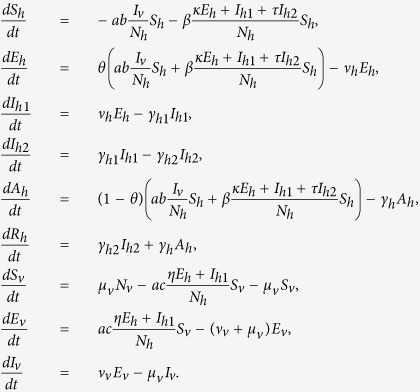


### Basic reproduction number

To understand the transmission dynamics, we analyzed the basic reproduction number 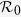, which is defined as the number of secondary cases produced by a single infective individual during his/her infectious period in an otherwise wholly susceptible population^23^. 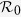 is a central concept in measuring the transmission of infectious diseases. For our Zika model, it is given by


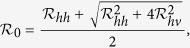


where


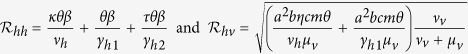


are the basic reproduction numbers of sexual transmission and vectorial transmission, respectively. In particular, 
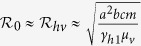
 provides the basic reproduction number of the classical Ross-Macdonald malaria model^23^ when sexual transmission and asymptomatic infection are rare, and intrinsic and extrinsic incubation periods of ZIKV in humans and mosquitoes are negligible. The necessary and sufficient condition for 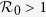 is 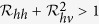, so the percentage of contribution by sexual transmission in 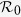 is 
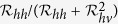
. 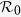 increases when the sexual transmission route is included. 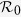 is increasing in *a*, *b*, *c*, *η*, *m*, *β*, *κ*, *τ*, *θ*, *ν*_*v*_, and decreasing in *ν*_*h*_,*γ*_*h*1_,*γ*_*h*2_,*μ*_*v*_. In particular, *β* (affected by safe-sex education), *m* (affected by vector control), and *a* (affected by bed net and insect repellent) are controllable parameters. Note that *β* is proportional to the fraction of unprotected sexual contact.

### Plug-and-play inference framework

The monthly simulated cases are obtained as 
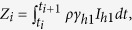
 where *ρ* denotes reporting ratio. We assume that the observed monthly cases (both confirmed and suspected) *C*_*i*_ is a random sample from a Negative-binomial (NB) distribution 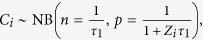 where *n* and *p* denote the size and probability of the NB distribution, and *τ*_1_ denotes an over-dispersion parameter which will be estimated. The mean and variance of the NB distribution are given by 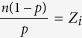 and 
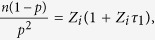
 respectively. The likelihood function for one country is 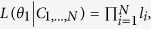 where *θ*_1_ denotes the parameter vector and *l*_*i*_ is the density associated with *C*_*i*_ and *Z*_*i*_. We apply the framework to three countries – Brazil, Colombia and El Salvador, which reported most cases continuously, and we denote the overall likelihood for the three countries as 
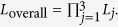


We use iterated filtering based plug-and-play inference framework^35^,^36^,^37^,^38^ to estimate *θ*_1_ via maximizing *L*_overall_. Also, we need the following assumptions to keep our model as simple as possible. First, many parameters in the model could be time-dependent. For simplicity, we focus on the scenario that the mosquito-human population ratio *m* is varying over time and use a cubic spline function to model *m*(*t*). Second, we assume that the three countries share common parameter values, except for country population size and initial conditions. Third, for parameters listed in Table 1, we use parameter values given there except for *m*, which we assume a cubic spline function of time with *n*_*m*_ parameters. Finally, with the two additional parameters, reporting rate *ρ* and over-dispersion parameter *τ*_1_, the number of parameters (*N*_*p*_) to be fitted is *n*_*m*_ + 2. We use Bayesian Information Criterion *BIC* = −2 log *L* + *N*_*p*_ log *N*_*d*_ to measure goodness-of-fit for models, where *N*_*d*_ denotes number of data points and *N*_*p*_ denotes number of free parameters.

## Additional Information

**How to cite this article**: Gao, D. *et al.* Prevention and Control of Zika as a Mosquito-Borne and Sexually Transmitted Disease: A Mathematical Modeling Analysis. *Sci. Rep.* **6**, 28070; doi: 10.1038/srep28070 (2016).

## Figures and Tables

**Table 1:** Parameter descriptions and ranges of the model.

	Description	Range	Value	Reference
*a*:	Mosquito biting rate. (Number of bites per mosquito per day)	0.3–1	0.5	39
*b*:	Transmission probability from an infectious mosquito to a susceptible human per bite. (Dimensionless)	0.1–0.75	0.4	39
*c*:	Transmission probability from a symptomatically infected human to a susceptible mosquito per bite. (Dimensionless)	0.3–0.75	0.5	40
*η*:	Relative human-to-mosquito transmission probability of exposed humans to symptomatically infected humans. (Dimensionless)	0–0.3	0.1	Assumed
*β*:	Transmission rate from symptomatically infected humans to susceptible humans. (Per day)	0.001–0.10	0.05	Assumed
*κ*:	Relative human-to-human transmissibility of exposed humans to symptomatic humans. (Dimensionless)	0–1	0.6	Assumed
*τ*:	Relative human-to-human transmissibility of convalescent to symptomatic humans. (Dimensionless)	0–1	0.3	Assumed
*θ* (%):	Proportion of symptomatic infections. (Dimensionless)	10–27	18	1
*m*:	Average ratio of mosquitoes to humans. (mosquitoes per human)	1–10	5	41
1/*ν*_*h*_:	Intrinsic incubation period in humans. (Days)	2–7	5	42
1/*ν*_*v*_:	Extrinsic incubation period in mosquitoes. (Days)	8–12	10	39, 43
1/*γ*_*h*1_:	Duration of acute phase. (Days)	3–7	5	42
1/*γ*_*h*2_:	Duration of convalescent phase. (Days)	14–30	20	12, 15
1/*γ*_*h*_:	Duration of asymptomatic infection. (Days)	5–10	7	Assumed
1/*μ*_*v*_:	Mosquito lifespan. (Days)	4–35	14	39, 40

**Table 2:** Parameter values and initial conditions used in Fig. 2.

Parameters	estimated	assumed range
*ρ*	0.012	[0, 1]
*τ*_1_	0.404	[0, ∞]
*m*_1_	0.001	[0, 20]
*m*_2_	9.525	[0, 20]
*m*_3_	13.100	[0, 20]
*m*_4_	8.129	[0, 20]
*S*_*h*,Brazil_	0.516	[0.5, 0.95]
*E*_*h*,Brazil_	0.000657	[9.79e-9, 1]
*I*_*h*1,Brazil_	0.000657	≡ *E*_*h*, Brazil_
*I*_*h*2,Brazil_	0.000657	≡ *E*_*h*, Brazil_
*A*_*h*,Brazil_	0.000657	≡ *E*_*h*, Brazil_
*S*_*v*,Brazil_		1-2e-4
*E*_*v*,Brazil_		1e-4
*I*_*v*,Brazil_		1e-4
*S*_*h*,Colombia_	0.705	[0.5, 0.95]
*E*_*h*,Colombia_	2.515e-07	[4.27e-8, 2.13e-7]
*I*_*h*1,Colombia_	2.515e-07	≡ *E*_*h*, Colombia_
*I*_*h*2,Colombia_	2.515e-07	≡ *E*_*h*, Colombia_
*A*_*h*,Colombia_	2.515e-07	≡ *E*_*h*, Colombia_
*S*_*v*,Colombia_		1-2e8
*E*_*v*,Colombia_		1e-8
*I*_*v*,Colombia_		1e-8
*S*_*h*,ElSalvador_	0.647	[0.5, 0.95]
*E*_*h*,ElSalvador_	8.076e-07	[3.25e-7, 1.628e-6]
*I*_*h*1,ElSalvador_	8.076e-07	≡ *E*_*h*, ElSalvador_
*I*_*h*2,ElSalvador_	8.076e-07	≡ *E*_*h*, ElSalvador_
*A*_*h*,ElSalvador_	8.076e-07	≡ *E*_*h*, ElSalvador_
*S*_*v*,ElSalvador_		1-2e-8
*E*_*v*,ElSalvador_		1e-8
*I*_*v*,ElSalvador_		1e-8

Note that parameters *ρ* and *τ*_1_ are used in the plug-and-play inference framework. The cubic spline interpolation functions *m*(*t*) = *a*_*i*_*t*^3^ + *b*_*i*_*t*^2^ + *c*_*i*_*t* + *d*_*i*_ in each interval *t* ∈ [*t*_*i*_, *t*_*i*+1_] with *i* = 1, 2, ···, *n*_*m*_ − 1 such that *m*(*t*_*i*_) = *m*_*i*_. In this interpolation, *m*_*i*_ are given and *t*_*i*_ (*i* = 1, 2, ···, *n*_*m*_) are the points that are uniformly distributed in the interval of one year.

**Table 3:** Numerical scenarios for Figs 4 and 6.

*β*	*m*	*a*	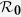	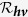	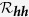	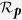 (%)	*z* (%)	*z*_*hv*_ (%)	*z*_*hh*_ (%)	*z*_*p*_ (%)
0.05	5	0.5	1.486	1.422	0.126	5.868	82.65	77.27	5.377	6.506
0	5	0.5	1.422	1.422	0	0	79.60	79.60	0	0
0.10	5	0.5	1.553	1.422	0.252	11.09	85.18	74.58	10.60	12.45
0.05	2.25	0.5	1.019	0.954	0.126	12.17	6.782	5.957	0.825	12.17
0	2.25	0.5	0.954	0.954	0	0	0	0	0	0
0.01	5	0.5	1.434	1.422	0.025	1.231	80.26	79.17	1.084	1.351
0.01	10	0.5	2.023	2.011	0.025	0.620	97.89	97.02	0.872	0.890
0.01	5	1	2.856	2.843	0.025	0.311	99.94	99.28	0.663	0.663
0.05	10	0.5	2.075	2.011	0.126	3.023	98.10	93.81	4.292	4.375
0.05	5	1	2.907	2.843	0.126	1.535	99.94	96.66	3.280	3.282

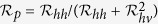
 and *z*_*p*_ = *z*_*hh*_/*z* are the percentages of contribution by sexual transmission in the basic reproduction number and attack rate, respectively, where *z*_*hv*_ and *z*_*hh*_ are the attack rates due to vectorial transmission and sexual transmission, respectively.
